# American Dental Degrees in England

**Published:** 1891-01

**Authors:** 


### Editorial.

### AMERICAN DENTAL DEGREES IN ENGLAND.

The American degree in England and on the Continent has been a subject of much controversy for many years. The disposition of this vexed question has in part been settled in Germany, Austria, Switzerland, etc., by a refusal to recognize any foreign degree, dental or medical. The same feeling seems to be growing in France, though we are not aware that it has, as yet, formulated into laws to prevent practice in that country.

In England the condition of things has been anomalous; but the tendency seems to be now to drive all Americans from practice in Great Britian.

The decision of the Medical Council was that the degrees of the Dental School of Harvard University and the Dental Department of the University of Michigan should alone be recognized. This decision has been repeatedly appealed from, but without any effect, and it remains in force at the present time. From what can be gathered through private sources, there has been added to this decision legal measures intended to drive out of the country all Americans employed as assistants to dentists. Tempted by a certain remuneration, quite a number of young graduates have accepted positions as assistants, but these must now return home. As far as they are concerned, this will, probably, end to their best interests.

It is understood that the Americans practising dentistry in London are fast leaving, and that those who remain will, with few exceptions, suffer professional and social ostracism.

It is not the place for one outside of a nationality to criticise the means taken to protect the class interests contained therein; but professional ties should, at least, be broader than governmental lines and free from the narrow prejudice of nationality.

The law of the British Council, if we may be permitted to give an opinion, has always had in it the elements of weakness. It has assumed to decide that, with two exceptions, all dental colleges in this country were unworthy of its high consideration. The two thus recognized, while excellent in themselves, have in no degree any superiority over those cast beyond the pale. The irritation which this action has caused does not concern us here; but the inconsistency of the measure is so marked that it is surprising that it has not been discovered by the ever-active English mind. The selection may have been a very wise one as to quality, but it must ever remain in history as a remarkable procedure. It is probable that our English cousins are beginning to realize the fact, from the disposition manifest to show no mercy in any direction.

If a suggestion may be permitted, it would seem to be the proper course, now that a change has been attempted, to make a clear sweep of all legal tolerance, and refuse to recognize any school on this side of the Atlantic. This would be, at least, consistent, but to what extent it may fulfil professional requirements may be a subject for future consideration.

The right of each nationality to put up the professional bars cannot be disputed. It is the right of self-protection; but in doing so, there can be no question but that the profession so using them lowers itself to the business standard of ethics, which means, strictly rendered, every one for himself. It is recognized that the areas of possibilities in securing a living are being narrowed yearly and are vastly more circumscribed in the old than in the new countries; yet it is not clear that the time has arrived for the institution of professional exclusiveness.

The average American has been so trained that it is impossible for him to understand why he cannot go anywhere in the wide world and settle, as he welcomes the wide world to come to him, and is, necessarily, startled at the police arrangements which metaphorically show him the door the moment he undertakes to invade the professional life of the people. All this is supremely selfish; but it is natural, and will come to the inhabitants of this country as soon as the lines have become so contracted as to bear heavily on the means of obtaining a livelihood.

All that can be, in reason, asked of our English *confrères* is that if they are determined to drive some Americans beyond British soil, not to stop there, but proceed to close the doors upon all and refuse to recognize any American school. This is the only course for the dental profession in England to accept; and while its adoption might work temporary injury to a few, it would, at least, place the profession of that country on tenable ground, where it does not, at present, stand.

